# Frequency of Anemia and Iron Deficiency in Ulcerative Colitis Patients in Clinical Remission as per the Patient-Reported Outcome (PRO-2) Score

**DOI:** 10.7759/cureus.90459

**Published:** 2025-08-19

**Authors:** Faiza S Ali, Bader F Zuberi, Tazeen Rasheed, Pawan Kumar, Sadia Sadaqat, Muhammad Rayyan, Amanullah Abbasi

**Affiliations:** 1 Medicine, Dow University of Health Sciences, Karachi, PAK; 2 Academic Coordination, Dow International Medical College, Karachi, PAK; 3 Medical School, Dow International Medical College, Karachi, PAK

**Keywords:** anemia of chronic inflammation, inflammatory bowel disease, iron deficiency anemia, non-anemic iron deficiency (naid), ulcerative colitis

## Abstract

Background

Iron deficiency anemia (IDA) is commonly present in patients with ulcerative colitis (UC) due to chronic blood loss, malabsorption, and chronic inflammation. Despite advances in the management of UC, the prevalence and clinical impact of IDA remain significant, potentially affecting disease progression, quality of life, and therapeutic response. Given the complex interplay between mucosal inflammation and systemic manifestations, early detection and optimal management of IDA in UC are crucial for improving patient outcomes. This study aimed to determine the prevalence, clinical characteristics, and treatment patterns of IDA in UC patients presenting to tertiary care centers in Karachi, Pakistan.

Methodology

This cross-sectional study was conducted in the Medical and Gastroenterology Department of Dr Ruth KM Pfau Civil Hospital Karachi and OMI Hospital from February 2024 and June 2025. Using non-parametric convenience sampling, adult patients (≥18 years) with a confirmed diagnosis of UC, based on clinical, endoscopic, and histopathological criteria, were enrolled consecutively. Data regarding demographics, duration and extent of disease, clinical activity (using the Patient-Reported Outcome (PRO-2) and Mayo scores), laboratory parameters (hemoglobin, serum ferritin, transferrin saturation, C-reactive protein), and treatment history were collected. IDA was defined as hemoglobin below the normal reference with evidence of depleted iron stores. Patients with other causes of anemia were excluded. Descriptive and inferential statistics were used to analyze the prevalence of IDA.

Results

In total, 216 patients met the inclusion criteria. The prevalence of IDA was found to be 76.9% among the study cohort. Of these, 27.8% were anemic with iron deficiency, 30.6% had anaemia of chronic inflammation with iron deficiency, and 18.5% had anemia of chronic inflammation without iron deficiency. Overall, 16.7% were non-anemic with iron deficiency, while 6.5% were non-anemic with adequate iron. Anemia was more prevalent in males compared to females.

Conclusions

IDA was a prevalent and under-recognized complication in patients with UC in the studied population. It was associated with greater disease activity and extent, yet remains inadequately managed in clinical practice. Proactive screening and timely initiation of appropriate iron replacement, particularly intravenous formulations, are recommended to optimize clinical outcomes, enhance quality of life, and support disease remission in UC. Future studies should explore barriers to optimal anemia management and evaluate the long-term benefits of integrated care approaches.

## Introduction

Inflammatory bowel disease (IBD) is a group of chronic immune-mediated inflammatory disorders that includes two disorders, i.e., Crohn’s disease (CD) and ulcerative colitis (UC). In a recent epidemiological survey, it was shown that the prevalence of IBD is rapidly increasing in the Asia-Pacific region [1]. Age-standardized incidence of UC in Asia ranges from 0.24 to 7.47 per 100,000 population [1]. Several reports from Asia, particularly from Japan, India, and Korea, have shown a higher prevalence of atypical UC distribution [2]. Although the exact etiology of IBD remains unidentified, there is consensus that both CD and UC result from an abnormal immune reaction to certain intestinal microbes in genetically predisposed hosts, with clinical flares of the diseases triggered and exacerbated by environmental factors [3]. Both, although encompassed under one heading, are quite different in their presentation, site, and pattern of involvement, treatment, and sequelae, with some similarities as well [4,5]. CD involves the full thickness of intestinal walls and can affect any section of the gut; however, it shows a predilection for the ileum and proximal colon [6]. UC is a mucosal disease affecting only the colon [7].

UC is a progressive disease that predisposes the patient to a high risk of complications if the inflammation in the colon is not effectively controlled. The main aim of treatment in UC is to achieve and maintain remission and prevent long-term complications such as colectomy, colorectal cancer, and disability by adequate control of inflammatory response [8]. But often overlooked is anemia and iron deficiency status in these patients, which is quite prevalent and adversely affects outcome [9]. Recently, the Selecting Therapeutic Targets in Inflammatory Bowel Disease (STRIDE-2) trial advocated for the goals toward altering the natural history of the disease, including clinical indices, biochemical biomarkers, and endoscopic and histological healing in UC. These recommendations have led to the concept of disease clearance, the ultimate goal in the management of UC [8]. This proposes total remission, including symptomatic and mucosal healing, including both endoscopic and histological healing, leading to molecular healing [10,11]. Patient-reported outcome measures (PROMs), including Patient-Reported Outcome (PRO-2), are now considered standard for assessing symptoms in UC. It comprises two subjective items of the Mayo score, i.e., stool frequency and rectal bleeding [12].

There is no report on the prevalence of UC in Pakistan, although there is consensus among gastroenterologists that it is now being diagnosed more frequently. A recent study reported a frequency of 11.52% of UC among patients undergoing sigmoidoscopy [13].

Anemia is a common complication of UC, with a prevalence reported with a wide variation of 9-73% in outpatients and 32-74% in admitted patients [4]. There are several probable causes of anemia in UC, including iron deficiency, vitamin B12 and folate deficiencies, and anemia of chronic inflammation (ACI), which was previously called anemia of chronic disease [14]. ACI is second to iron deficiency anemia (IDA) in terms of prevalence [15]. Recent evidence suggests that ACI and IDA sometimes co-exist, such as in patients on dialysis, congestive cardiac failure, chronic obstructive lung disease, obesity, and chronic liver disease [16]. Proper differentiation of the type of anemia is necessary for its adequate treatment in UC.

Correcting iron deficiency and subsequent anemia improves the quality of life of patients with UC, while administering iron in ACI could be harmful [17]. In UC, anemia could be due to both iron deficiency and ACI and should be differentiated for proper management. While hemoglobin, mean corpuscular volume, and transferrin saturation (TSAT) are decreased in both types of anemias, the differentiation factors are that ferritin and hepcidin are elevated in ACI, while they are decreased in IDA. Detection of iron deficiency in UC will benefit patients as iron is not only essential for erythropoiesis but is also required for normal functions of the immune system via neutrophils and macrophages [17]. Once detected, iron deficiency can be corrected by iron replacement by either intravenous or oral routes.

This study aimed to determine the frequency of anemia and iron deficiency in UC patients in clinical remission.

## Materials and methods

This cross-sectional study was conducted in the Medical and Gastroenterology Department of Dr Ruth KM Pfau Civil Hospital Karachi, from February 2024 to June 2025, following IRB approval vide letter (approval number: IRB-13330/DUHS/Approval/2024/32 dated February 12, 2024). Individuals with UC of any gender and aged between 18 and 60 years were selected through non-parametric convenience sampling after obtaining informed written consent. The PRO-2 scoring system was used, and only patients with a total score of 0, indicating clinical remission, were enrolled. Patients with hemorrhoids, malignancies, peptic ulcer disease, hemoglobinopathies, intestinal resections, tuberculosis, pregnancy, vitamin B12 and folate deficiencies, or those already receiving iron therapy were excluded.

The sample size was determined using the previously reported local frequency of 27.6% [4] for anemia in UC patients, with power set at 95% and α at 0.05. The sample size was calculated as a minimum of 59 patients using the PASS software. We exceeded the calculated sample size to add robustness to the study findings.

PRO-2 scores were determined based on the following two parameters: stool frequency: pathological if ≥4 stools/day (1 point); and rectal bleeding: pathological if traces of blood are present (1 point). The PRO-2 score was calculated as the sum of the two sub-scores.

After obtaining informed consent, each selected patient underwent a routine clinical examination, and their demographic information was documented. A 10 mL blood sample was collected by a phlebotomist and placed in red and green top tubes for laboratory analysis, which included complete blood count, ferritin, iron, and total iron-binding capacity (TIBC). TSAT was calculated using the following formula: TSAT = (Iron/TIBC) × 100.

**Figure 1:**
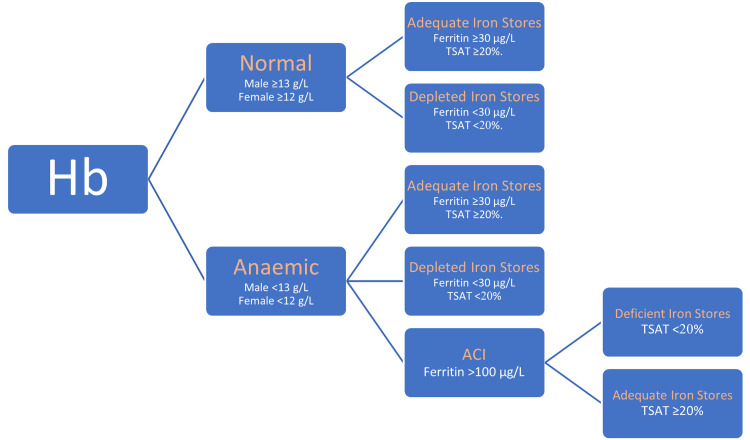
Patient classification according to anemia and iron status. ACI = anemia of chronic inflammation; Hb = hemoglobin; TSAT = transferrin saturation

Anemia was defined as hemoglobin levels below 13 g/L in males and below 12 g/L in females. Iron deficiency was identified if ferritin was less than 30 µg/L and/or TSAT was below 20% [18]. ACI was diagnosed in anemic patients with ferritin levels above 100 µg/L [19]. These patients were further categorized as iron-deficient if their TSAT was below 20%, and as having adequate iron stores if their TSAT was 20% or higher. Classification of patients followed the algorithm presented in Figure 1.

The frequency of anemia and its types was calculated. The frequency of iron deficiency was determined and cross-tabulated for the presence or absence of anemia. The chi-square test was used for frequency comparison, with significance set at p-values ≤0.05.

## Results

A total of 216 patients participated in the study, including 122 (56.5%) males and 94 (43.5%) females. Mean age ± standard deviation (SD) of males was 33.36 ± 8.45 years, while that of females was 33.51 ± 8.73 years. There was no significant difference in age between genders, as tested by Student’s t-test t (214) = -0.127, p = 0.899. Notable differences were noted between genders regarding hemoglobin and iron indices, as summarized in Table 1.

**Table 1:** Comparison of hemoglobin, Iron, TIBC, ferritin, TSAT levels between genders by Student’s t-test. Student’s t-test was used, with significance set at <0.05. TIBC = total iron-binding capacity; TSAT = transferrin saturation

Parameter	Gender	Mean	±SD	t	Sig.	95% confidence interval	
						Lower	Upper
Hemoglobin (g/dL)	Male (14–18 g/dL)	11.42	1.87	8.159	<0.001	1.484	2.429
	Female (12–16 g/dL)	9.46	1.58				
Serum iron (µg/dL)	Male (75–150 µg/dL)	56.82	30.29	2.936	0.004	3.556	18.082
	Female (60–140 µg/dL)	46	21.56				
TIBC (µg/dL)	Male (171–505 µg/dL)	354.33	54.12	-3.833	<0.001	-35.685	-11.445
	Female (149–492 µg/dL)	377.89	28.43				
Ferritin (µg/L)	Male (12–300 ng/dL)	71.3	43.39	2.518	0.013	3.423	28.102
	Female (10–150 ng/dL)	55.53	48.36				
TSAT (%)	Male (20–50%)	17.85	13.43	3.472	<0.001	2.284	8.283
	Female (20–50%)	12.56	6.95				

Of the 216 patients, 50 (23.1%) had adequate hemoglobin levels. Among these, 14 (6.5% of the total) patients exhibited both normal hemoglobin and iron profiles, while 36 (16.7%) were iron-deficient despite having hemoglobin within the normal range. This demonstrates that a considerable proportion of patients who were not anemic still had iron depletion. Anaemia was identified in 166 (76.9% of the cohort) patients. The prevalence and distribution of iron deficiency and types of anemia are presented in Table 2.

**Table 2:** Hemoglobin and iron status by gender and comparison by the chi-square test. Pearson’s chi-square test was used, with significance set at <0.05. 0 (0.0%) cells have an expected count <5. The minimum expected count is 6.09. ACI = anemia of chronic inflammation

	Gender		Total	df	Chi-square	Sig.
	Male	Female	n (%)			
Non-anemia: adequate iron	12 (5.6)	2 (0.9)	14 (6.5)	4	39.741	<0.001
Non-anaemia: depleted iron	28 (13.0)	8 (3.7)	36 (16.7)			
Anemia: depleted iron	16 (7.4)	44 (20.4)	60 (27.8)			
ACI: depleted iron	42 (19.4)	24 (11.1)	66 (30.6)			
ACI: adequate iron	24 (11.1)	16 (7.4)	40 (18.5)			
Total	122 (56.5)	94 (43.5)	216 (100.0)			

Key findings are as follows: (1) of all participants, only 6.5% (n = 14) had both non-anemic and iron-replete profiles; (2) iron deficiency without anemia was identified in 16.7% (n=36) of patients; (3) the overwhelming majority, 76.9% (n = 166), were anemic; and (4) among anemic patients, 27.8% (n = 60) had depleted iron stores, 30.6% (n = 66) had ACI along with iron depletion, and 18.5% (n = 40) had ACI with adequate iron stores.

These results indicate that both overt and subclinical iron deficiency are prevalent among patients with UC in remission, with significant gender-based disparities observed in hematological and iron indices.

## Discussion

The present study provides valuable insights into the prevalence and nature of anemia and iron deficiency among patients with UC in clinical remission. The persistence of iron deficiency among patients with UC in remission can be attributed to a complex interplay of factors that extend beyond active gastrointestinal bleeding. Even in the absence of overt symptoms, chronic inflammation may subtly impair iron metabolism by elevating hepcidin levels [19], a regulatory hormone that restricts iron absorption from the gut and limits mobilization from stores. Additionally, previous episodes of blood loss, whether from active disease flares or microscopic bleeding undetected during remission, can leave patients with depleted iron reserves that are slow to recover. Malabsorption also plays a critical role [20]. Damage to the intestinal mucosa, even if healed, may not fully restore the gut’s capacity to absorb dietary iron, especially in the proximal colon and terminal ileum, where iron uptake is significant. Dietary restrictions or poor intake, sometimes adopted to manage symptoms or due to food intolerances, can further contribute to inadequate iron supply. Moreover, repeated inflammation may cause ongoing, low-grade iron loss and disrupt the delicate balance required for optimal erythropoiesis.

Physiological factors add further complexity; women, for example, may experience compounded iron loss due to menstrual blood loss, amplifying their susceptibility to deficiency. The interplay between these mechanisms means that iron deficiency can persist, or even silently progress, during periods when UC appears quiescent. This multifactorial nature underscores the necessity for proactive monitoring and individualized management in this patient group.

Notably, in our study, 76.9% of the patients were anemic, underscoring that anemia remains a significant issue in this population even during periods of clinical quiescence. This finding is consistent with existing literature, which highlights that anemia in UC is multifactorial, frequently persisting despite remission of gastrointestinal symptoms. In a study from Pakistan, anemia prevalence was reported at 51.2% in patients with UC in remission [9]. A study from India reported an anemia prevalence of 53.1% in patients with UC in remission [21]. The frequency of anemia in our study was higher than reported earlier.

A further important observation is the distribution of iron status among both anemic and non-anemic patients. Among those with normal hemoglobin levels, a substantial proportion (16.7%) displayed depleted iron stores, while in this group, only 6.5% had adequate iron. This suggests that iron deficiency can be present sub-clinically and may precede the development of overt anemia in UC patients. Such iron depletion, even in the absence of anemia, may contribute to symptoms such as fatigue and impaired quality of life [22-24], emphasizing the need for routine iron status screening in this cohort. Correction of iron stores results in improvement in these symptoms [22].

The gender-based analysis revealed statistically significant differences in hemoglobin, iron, TIBC, ferritin, and TSAT levels, with female patients displaying lower hemoglobin and iron indices than males. This aligns with established observations that females, owing to physiological factors such as menstruation in addition to chronic disease, are at an increased risk for iron deficiency and anemia [25]. This makes the case for targeted screening of females for iron stores in UC remission.

When categorizing anaemia, the study distinguished IDA and ACI, as well as their overlap with iron depletion. Overall, 30.6% of those with ACI also had iron depletion, and 18.5% of ACI cases retained adequate iron stores. These nuanced findings reinforce that anemia in UC is not a uniform entity, but rather a spectrum, including classic iron deficiency, chronic inflammation, and mixed patterns. Such differentiation is clinically important, as the approach to treatment, be it iron supplementation or addressing ongoing inflammation, should be tailored accordingly.

When considering the optimal strategy for correcting iron deficiency in patients with UC, the choice between oral and intravenous iron supplementation depends on several clinical factors. Oral iron remains the first-line therapy for many patients due to its accessibility, cost-effectiveness, and ease of administration. It is generally suitable for individuals with mild iron deficiency, preserved intestinal absorption, and few or no ongoing symptoms of active disease. However, the efficacy of oral iron can be limited in UC patients by diminished mucosal absorption, gastrointestinal intolerance, or ongoing inflammation that impedes uptake, often resulting in suboptimal replenishment of iron stores. Intravenous iron, in contrast, is indicated for patients who have moderate-to-severe iron deficiency, intolerance or non-responsiveness to oral therapy, active disease with compromised gastrointestinal absorption, or those who require rapid correction of iron deficits. Intravenous iron bypasses the gastrointestinal tract, ensuring efficient and predictable repletion of iron stores, and is particularly advantageous in cases complicated by chronic inflammation, where hepcidin-mediated blockade of intestinal iron uptake is prevalent [26].

Taken together, the high prevalence of both overt and latent iron deficiency in UC patients in remission suggests that gastrointestinal mucosal healing does not necessarily equate to the restoration of adequate iron stores or red cell indices. This underscores the importance of comprehensive assessment, including hemoglobin, ferritin, and TSAT, in the routine follow-up of UC patients, even those in symptomatic remission.

In summary, these results highlight the need for vigilance regarding anemia and iron deficiency among UC patients in remission. Early identification and targeted management and use of relaxation therapy and exercise may improve patient outcomes, prevent complications, and enhance overall quality of life [27].

Limitations

While the present study offers important insights into the prevalence and characteristics of anemia and iron deficiency among patients with UC in clinical remission, several limitations must be acknowledged. The findings are based on a specific cohort, which may not fully represent the broader population of UC patients in remission. The relatively small or single-center sample may limit the generalizability of the findings to other demographic groups or clinical settings. The cross-sectional design of the study restricts the ability to draw conclusions about causality or the progression of iron deficiency and anemia over time. Prospective studies would be valuable to assess how iron status fluctuates with disease activity and remission. Although the study considered gender and iron indices, other confounding variables such as dietary iron intake, coexisting medical conditions, medication use, and genetic factors were not exhaustively explored, which could influence iron status and anemia prevalence.

## Conclusions

This study underscores the persistent and multifaceted nature of anemia and iron deficiency among patients with UC in clinical remission. Despite the absence of overt gastrointestinal symptoms, a substantial proportion of this population continues to experience both overt and subclinical iron depletion, significantly impacting their quality of life. The findings highlight the necessity for routine, comprehensive evaluation of iron status, including hemoglobin, ferritin, and TSAT levels, regardless of symptomatic remission status. Furthermore, the observed gender disparities and diverse patterns of anemia reinforce the importance of individualized assessment and management strategies. Addressing these issues proactively has the potential to improve clinical outcomes and the overall well-being of UC patients, emphasizing the critical role of ongoing vigilance in the care of this patient group.
